# Cognitive Profiles of Children with Low Motor Performance: A Contribution to the Validation of the WPPSI-IV

**DOI:** 10.3390/children9050619

**Published:** 2022-04-26

**Authors:** Julia Jascenoka, Franziska Walter

**Affiliations:** 1 Department of Educational Psychology, Helmut-Schmidt-University/University of the Federal Armed Forces, 22043 Hamburg, Germany; 2 Department of Medicine, Medical School Hamburg, 20457 Hamburg, Germany; franziska.walter@medicalschool-hamburg.de

**Keywords:** movement abilities, Developmental Coordination Disorder (DCD), cognitive profiles, WPPSI-IV, LoMo 3-6, intelligence

## Abstract

(1) Background: Developmental Coordination Disorder (DCD) is a common developmental disorder of preschool age. Children often show cognitive deficits in addition to motor problems. Various studies point in particular to problems in visual perception, working memory and processing speed. In this context, it is investigated whether the Wechsler Preschool and Primary Scale-IV (WPPSI-IV) is a suitable instrument for mapping these deficits in a valid and economical way. (2) Methods: The WPPSI-IV profiles of children with DCD (n = 12), below-average motor performance (n = 22) and a control group (n = 32) were compared. (3) Results: Children with DCD achieved significantly poorer test performance in the primary indices Verbal Comprehension, Visual Spatial, Processing Speed and Full Scale compared to a control group. Children with below-average motor skills, on the other hand, do not differ from the children in the control group. (4) Conclusions: The WPPSI-IV is a suitable instrument for diagnosing cognitive deficits in the context of DCD. The Fluid Reasoning and Verbal Comprehension indices should be used as a supplement to assess cognitive performance levels.

## 1. Introduction

Developmental Coordination Disorder (DCD) is among the most common developmental disorders of preschool and primary school age and is symptomatically characterized by delayed achievement of key motor milestones and a significant degree of motor clumsiness [1,2]. Affected children are most notable for deficits in fine and gross motor coordination, lack of balance and considerable handwriting problems [2], although these cannot be attributed to neurological, sensory or cognitive impairments [3,4]. Recent studies have shown that everyday life is not only complicated by the motor deficits themselves, but that most children are also burdened by psychosocial and cognitive problems [2]. With a prevalence of about 5%, DCD occurs in a wide range of cultural groups [5,6,7,8]. Boys are affected more often than girls [9]. Although the disorder is a widespread phenomenon, DCD is often not diagnosed (in time) in everyday clinical and educational practice or is limited to the level of motor function [10,11]. In view of the complex constellation of symptoms, however, a multimodal diagnosis by a multiprofessional team would be required in order to initiate comprehensive therapeutic measures at an early stage and to positively influence the developmental prognosis of children with motor disorders in the long term [3,12].

### 1.1. Cognitive Profiles of Children with DCD or Risk of DCD

DCD is often accompanied by various cognitive deficits [13,14]. DCD appear to be strongly associated with lower performance in the areas of visual perception, attention and memory. Deficits in visual perception have been shown to manifest in eye–hand coordination, visual-perceptual performance and visual–motor integration [11]. Other studies have consistently found abnormalities in the visual perceptual abilities of shape constancy, spatial relations, visual memory, visual discrimination and figure-ground discrimination [15,16,17]. Asonitou, Koutsouki, Kourtessis and Charitou [18] additionally found out that children with DCD exhibit deviant selective attention and problems in attentional control. According to Leonard, Bernardi, Hill and Henry [12,19], abnormalities in various executive functions, such as auditory working memory, inhibition, planning ability and fluency, can also be observed in association with DCD. Michel, Kauer and Roebers [20] in their study of children with risk of DCD (PR > 50 in an international motor skills test), revealed that, on average, they performed more slowly in the cognitive-executive domains than children without motor problems, but with the same level of accuracy. Chen, Tsai, Hsu, Ma, and Lai [21] also demonstrated a lower everyday memory performance for children with DCD compared to a control group with no motor deficits, with the largest group differences in auditory and visual memory domains.

### 1.2. Measuring Cognitive Profiles in Children with Movement Disabilities Using Intelligence-Testing

In 2019, the European Children Academy repeatedly published evidence-based diagnostic and therapeutic recommendations to establish optimal standards of care for patients with DCD [3]. In addition to the comprehensive clarification of motor developmental deficits, the assessment of cognitive performance also plays an important role in the diagnostic process, clarifying whether it is a circumscribed motor developmental deficit and not much more a global developmental delay or reduced intelligence [4]. The description of the cognitive strengths and weaknesses of a child with DCD is also important for the planning of appropriate intervention strategies [22]. 

Due to their design features, classical intelligence tests offer the possibility to systematically and economically examine a comprehensive spectrum of cognitive performance (e.g., language performance, visual perceptual performance, memory performance, attention performance, etc.). The total duration of the test is about 60 to 90 min, which is significantly shorter than completing several tests of distinct cognitive dimensions. In addition, based on “golden diagnostic standards”, it is possible to give concrete recommendations for everyday clinical practice [23].

Intelligence test procedures based on the Wechsler scales are internationally established and enable a differentiated description of cognitive performance profiles. The WPPSI_IV is a comprehensive individual test procedure for preschool children, which is frequently used in research and practice. Cognitive performance in the dimensions of visual perception, working memory, fluent reasoning, verbal comprehension and processing speed are assessed using up to 15 subtests. It can therefore be assumed that the initially formulated demand for early detection of cognitive deficits in children with DCD can be met with the Wechsler Preschool and Primary Scale (WPPSI-IV) [24,25]. Compared to the previous version, the WPPSI-III [26], the current WPPSI-IV demonstrated a new factor-structure [23]. The WPPSI-III still allowed for a subdivision into Verbal-IQ and Performance-IQ as well as the formation of the indices’ General Language Scale and, for children from four years, the index Processing Speed. For WPPSI-IV, two test versions, such as in WPPSI-III, are available. The longer test version, which was designed for children aged four years to seven years now comprises five primary indices (Verbal Comprehension Index, Visual Spatial Index, Fluid Reasoning Index, Working Memory Index as well as Processing Speed Index), four ancillary indices (Vocabulary Acquisition Index, Nonverbal Index, General Ability Index as well as Cognitive Proficiency Index) as well as an overall scale (Full-Scale IQ). These new indices, as well as revised and supplementary subtests, are intended to contribute to a more differentiated description of the performance of kindergarten and pre-school children across various cognitive dimensions. In the context of diagnosing children with DCD, the changes in the processing mode of the Processing Speed index are particularly noteworthy in terms of both content design and test item implementation: In order to reduce the influence of motor skills, target stimuli are now no longer crossed out with a pencil, but marked with a stamp [27]. The addition of subtests assessing (visual) working memory skills to the WPPSI-IV also offers advantages in the diagnostic process of DCD: The cited results of Leonard et al. [19] and Chen [21] et al. underline the benefits of the routine testing of working memory skills. A further advantage relates to the new factor structure of WPPSI-IV in order to that of WPPS-III. The new second-order five-factor model for the longer test version (age group 4:0–7:7) of the WPPSI-IV contains, among others, the change that the former index score Performance-IQ of the WPPSI-III is divided into the new index scores Visual-Spatial Index and Fluid Reasoning Index of the WPPSI-IV. Based on the new factor structure deficits in different cognitive abilities in children with diagnosis DCD can be tested more specifically. 

To the best of our knowledge, there are currently no studies that describe specific intelligence profiles of children with motor problems in the WPPSI-IV. It needs to be clarified to what extent the problems described in the literature in various cognitive domains can effectively be demonstrated by performances in the WPPSI-IV. This particularly applies to visual perception, (visual) working memory and processing speed. Studies on the WPPSI-III showed that children diagnosed with DCD achieved significantly lower test scores than participants of the control group parallelized by age and sex, both at the Full-Scale IQ and in the Verbal-IQ and Performance-IQ [28,29]. At subtest level, both studies describe significant effects of motor deficits on the subtest Block Design, which measures visual and motor skills to a large extent. Furthermore, Jascenoka et al. [29] also confirmed suspected performance differences in Processing Speed (Symbol Search and Coding); both subtests require not only visuo-motor coordination but also cognitive processing speed, visual short-term memory and attention skills. Best test performance was achieved by children with DCD in the subtest Picture Concepts [28,29], which primarily requires categorical, abstract thinking. Both studies recommend using this subtest to estimate the actual intelligence level of children with DCD. The authors of the WPPSI-IV also point out (see test manual, p. 28) that motor limitations can lead to an underestimation of a child’s actual intellectual abilities and that fine motor developmental deficits lead to low test performance in those tasks with fine motor demands as well as time limitations. According to the authors, this is especially true for the subtests Block Design, Zoo Location, and Object Assembly, which is why it is recommended that the intelligence level of children with severe motor impairments be assessed using only the primary indices Verbal Comprehension and Fluid Reasoning [24].

### 1.3. Aims

The aim of the present study is to investigate whether the WPPSI-IV is a valid instrument in the diagnostic process of DCD to identify support needs in different cognitive performance domains as well as for estimating the “actual” intelligence level (*Question 1*). Few studies (e.g., Michel et al. [20]) examine the cognitive performance of children with lower motor performance, but it cannot be classified as DCD. Therefore, not only children who meet the diagnostic criteria of a DCD should be examined, but also children with below-average motor performance (PR < 50) in a motor development test. If similar tendencies in the cognitive performance profiles of the WPPSI-IV already become apparent with slightly deviating motor skills (PR < 50 in a motor development test), this provides an important clue for offering low-threshold support services to children with deficits in motor skills at an early stage (e.g., in the kindergarten and home environment) (*Question 2*). In this way, the motor and cognitive skills of future school-age children can be strengthened and the prognosis for a good start at school can be improved.

Based on the cited research results, it is assumed that children with DCD showed a lower score in visual perception, which negatively effects the subtests of the primary index Visual Spatial (subtests: Block Design, Object Assembly) [11,15,16,17,28,29] compared to the control. Furthermore, children with DCD also demonstrated reduced working memory performance with lower test performance in the Working Memory index (subtests: Picture Memory, Zoo Locations) [18,19]. The extent to which DCD influences test performance in the newly developed subtests Bug Search and Cancellation (index Processing Speed) remains an open question and will be examined in the context of this study. The results of a study by Sumner, Pratt and Hill on WISC-IV [30] indicate that children with DCD had no differences in the Index Processing Speed after controlling for manual dexterity compared to a control group with no motor problems. It thus seems possible that the revision of the subtests of the index Processing Speed effectively reduces the influence of fine motor deficits, so that there are no differences in performance between children with and without DCD, or that these differences are small. Whether the recommendations of the WPPSI-IV authors regarding the estimation of the intelligence level of children with movement disabilities on the basis of the primary indices Verbal Comprehension and Fluid Reasoning can be empirically proven and thus contribute to confirming the diagnosis of DCD (exclusion criterion of intelligence impairment) is to be tested by the present data.

## 2. Materials and Methods

### 2.1. Data Collection and Sample

In the present study, 66 children (32 girls, 34 boys) were examined regarding their cognitive and motor abilities. The WPPSI-IV was used to test intelligence performance; the motor development status was checked with the German LoMo 3-6 motor test (Leistungsinventar zur objektiven Überprüfung der Motorik von 3-bis 6-Jährigen [31]). The LoMo 3-6 was specifically developed for the diagnosis of DCD; validation studies with the Movement-ABC-2 (an internationally recognized motor test procedure) [32] exhibit significant correlations for the total motor score (r = 0.511). The participating children were between 48 and 81 months (M = 61.73; SD = 8.19) old. The recruitment of the sample took place within the norming and validation projects for the German adaptation of the Wechsler Preschool and Primary Scale of Intelligence-Fourth Edition (WPPSI-IV [24]) as well as the Leistungsinventar zur objektiven Überprüfung der Motorik von 3-bis 6-Jährigen (LoMo 3-6 [31]). The following exclusion criteria were defined: insufficient knowledge of the German language, participation in an intelligence test within the last six months, significant limitations of the upper and lower extremities (e.g., cerebal palsy), taking medication that can influence test performance (e.g., anticonvulsants), radiation treatment of the central nervous system, and the presence of a diagnosed physical, neurological or mental illness that significantly affects the child’s ability to conduct (e.g., stroke, brain tumour, epilepsy). To answer the main questions of this study (*Question 1*: Can cognitive deficits expected in the context of DCD be represented by the WPPSI-IV?; *Question 2*: Do cognitive performance profiles differ depending on the severity of motor impairments?), the total sample was divided into three subsamples based on the test performance in the LoMo 3-6 (given in percentile ranks). This categorization was based on the recommendations of the LoMo 3-6 manual. A percentile rank < 25 indicates the presence of a DCD or the risk (DCD) of such a disorder (n = 12) (diagnostic criterion I according to DSM- V [4]); a percentile rank of 26 to 50 indicates a motor performance that is below average (BA) but not in need of therapy (n = 22). A percentile rank of >50 indicates normal abilities (n = 32) (control group). The additional parent questionnaire on everyday motor activities indicated, furthermore, that the children in the DCD or at risk of DCD subsample also showed impairments in everyday activities (diagnostic criterion II according to DSM- 5 [4]) and the children did not show any sensory or neurological deficits (diagnostic criterion III according to DSM-5 [4]). A comprehensive description of the sociographic characteristics of the total and partial samples is provided in Table 1.

### 2.2. Procedure

The tests were administered in various kindergartens in northern Germany (federal states of Lower Saxony, Bremen and Hamburg) and at the Centre for Clinical Psychology and Rehabilitation at the University of Bremen. The tests were administered by extensively trained psychology students and psychologists. In order to ensure the optimal concentration ability of the children, both procedures were usually performed on two different days. The interval between intelligence and motor testing was a maximum of three weeks for about 70% of the children (min = 0 days, max = 138 days). However, if both test procedures were completed on one day, the WPPSI-IV was started without exception. Written consent was obtained from all parents for the scientific use of the data material. All parents were informed about the confidential and anonymous handling of their personal data and the data of their children. The WPPSI-IV test took about 60 to 90 min to complete, depending on the child’s ability. The LoMo 3-6 motor skills test took about 35 to 45 min to complete.

### 2.3. Measuring Procedure

#### 2.3.1. Wechsler Preschool and Primary Scale-Fourth Edition (WPPSI-IV, Wechsler, 2018)

The WPPSI-IV [24] is an individual test procedure for kindergarten and preschool children between 2:6 and 7:7 years. Two different versions are available for children from 2:6 to 3:11 and from 4:0 to 7:7 years; in the present study, the version for children from 4:0 years was used. In addition to an overall IQ (M = 100, SD = 15), cognitive performance can be described in a more differentiated way using primary and ancillary index scores (see Section 1.2). A total of ten primary subtests are available for calculating the primary index scores. Two subtests are assigned to each scale. Verbal Comprehension is formed from the subtests Information and Similarities. The index Visual Spatial is calculated from the subtests Block Design and Object Assembly. Fluid Reasoning consists of the Matrix Reasoning and the subtest Picture Concepts. The subtests Picture Memory and Zoo Locations form the index Working Memory. Furthermore, Bug Search and Cancellation are used to determine processing speed. Six primary subtests are needed to determine total IQ (Information, Similarities, Block Design, Matrix Reasoning, Picture Concepts and Bug Search). The present study only includes primary index scores and subtests, as these have a great practical relevance and an implementation of all 15 available subtests is less common in clinical practice. The reliability for the Full-Scale IQ is r = 0.95. Intercorrelation studies, factor-analytical as well as correlation studies with other psychometric tests are available to demonstrate validity.

#### 2.3.2. Leistungsinventar zur objektiven Überprüfung der Motorik von 3-bis 6-Jährigen (LoMo 3-6, Jaščenoka and Petermann, 2018)

The LoMo 3-6 [31] is used to assess the motor performance of kindergarten and preschool children. Two test versions are available for younger (3:0 to 4:5 years) and older children (4:6 to 6:11 years). Younger children are presented with 22 items, a shortened and slightly modified version of the full test version (32 items). Only Version B was used in the present study. A total score is calculated from all test items, which represents a child’s level of motor development in the areas of fine motor skills, total body coordination, bilateral coordination, object control as well as strength and endurance. The retest reliability of the procedure for the raw values of the total motor score can be estimated as stable with r = 0.84.

### 2.4. Statistical Analyses

All calculations were carried out with the statistical package IBM SPSS 27 Statistics. After entering the data sets, plausibility checks were carried out to ensure data quality. To investigate the influence of the group factor motor performance (DCD or risk of DCD (PR < 25), Below Average (BA, PR 26–50), Control Group (CT, PR > 50)) on intelligence performance in the WPPSI-IV, group comparisons (one-factor ANOVA) were performed. The prerequisites of normal distribution (Shapiro–Wilk) (with a few exceptions in the CT group), homoskedasticity (Levene’s test) as well as sufficiently large samples (at least as many cases in each factor level as dependent variables) were given. In addition, there was no multicolinearity (r < 0.85) [33] or univariate or multivariate outliers (Mahalanobis distance, *p* > 0.001). Post-hoc analyses were conducted to compare individual groups (*t*-test) (see Appendix B). In addition, effect sizes (η^2^) according to Cohen [34] (η^2^ > 0.01 = small, η^2^ > 0.06 = medium, η^2^ > 0.13 = large) were calculated. Since the requirement of a sufficiently large sample is only minimally fulfilled (factor level DCD n = 12), all results were verified by means of non-parametric procedures (Kruskal–Wallis or Mann–Whitney U) (see Appendix A).

## 3. Results

### 3.1. Descriptive Statistics and Group Comparisons for the Primary Indices and Full-Scale IQ of the WPPSI-IV

**Figure 1:**
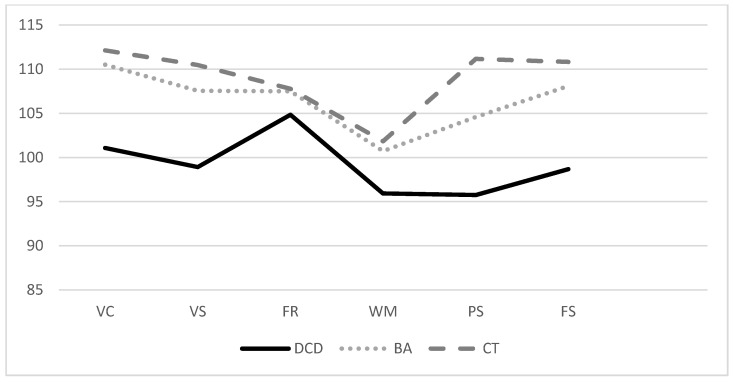
Mean scores of the primary WIPPSI-IV index scores and the FSIQ as comparison between groups. Note: VC = Verbal Comprehension, VS = Visual Spatial, FR = Fluid Reasoning, WM = Working Memory, PS = Processing Speed, FS = Full Scale-IQ.

Table 2 illustrates the group differences in different primary index scales of WPPSI-IV between participants with DCD, children with below average motor performance (BA), and a control group (CT). Examination of the descriptive statistics indicates that children with DCD or at risk of DCD achieve lower test performance in all five primary indices than children with below-average and above-average motor performance. Children with DCD indicated the lowest score in the index Processing Speed Index and the highest scores in the index Fluid Reasoning Index. Children without DCD, on the other hand, reveal a different profile: the best test performance was in the index Verbal Comprehension (BA: M = 110.50, SD = 13.88; CT: M = 112.13, SD = 11.83), and the lowest score was seen in the index Working Memory (BA: M = 100.73, SD = 13.41; CT: M = 101.84; SD = 10.68) (see also Figure 1). The statistical comparison of all three groups by means of one-factor ANOVA exhibits significant group differences for the indices Verbal Comprehension, Visual Spatial, Processing Speed as well as the Full-Scale IQ (Table 2). The effects can be classified as medium (Verbal Comprehension: η^2^ = 0.101, Visual Spatial: η^2^ = 0.115) to large (Processing Speed: η^2^ = 0.170; Full Scale: η^2^ = 0.164) (Table 2). Subsequent post-hoc procedures revealed significant differences in performance between children in the DCD and CT group for the indices Verbal Comprehension (*p* = 0.008, d = −0.94), Visual Spatial (*p* = 0.006, d = −0.97), Processing Speed (*p* = < 0.001; d = −1.25) and Full-Scale IQ (*p* = 0.002, d = −1.14). In addition, significant deviations between the DCD and BA groups can be found for the Full-Scale IQ (*p* = 0.018, d = −0.89); children with DCD achieved a mean score in the Full-Scale IQ of M = 98.67 (SD = 12.21), while children in the BA group had a mean score of M = 108.09 (SD = 9.59). The test results of the two groups BA and Control neither differ in the primary indices (Verbal Comprehension: *p* = 0.646, d = −0.12, Visual Spatial: *p* = 0.371, d = −0.20, Fluid Reasoning: *p* = 947, d = −0.02., Working Memory: *p* = 0.735, d = −0.09., Processing Speed: *p* = 0.091, d = −0.47) nor in the Full-Scale IQ (*p* = 0.278, d = −0.30) (see Appendix B).

### 3.2. Descriptive Statistics and Group Comparisons for the Subtests of the Primary Indices of WPPSI-IV

**Figure 2:**
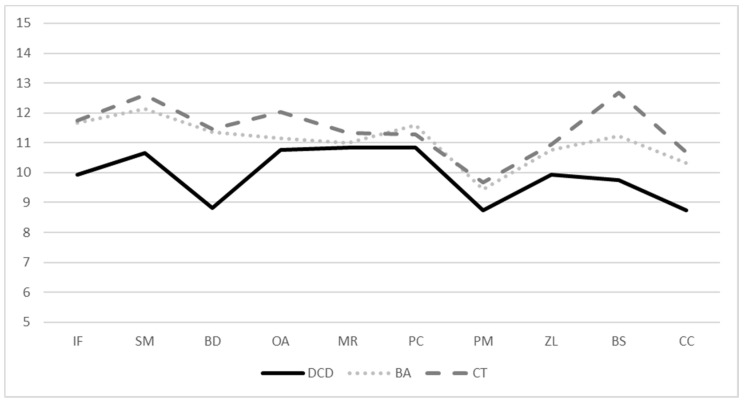
Mean scores of the ten primary WIPPSI-IV subtests (scaled scores). Note: IF = Information, SM = Similarities, BD = Block Design, OA = Object Assembly, MR = Matrix Reasoning, PC = Picture Concepts, PM = Picture Memory, ZL = Zoo Locations, BS = Bug Search, CA = Cancellation.

A more differentiated examination at subtest level indicates the lowest performance for children with DCD in the subtests Block Design (M = 8.83; SD = 2.21) and Picture Memory (M = 8.75; SD = 2.83), while the highest test scores were achieved in the Matrix Reasoning (M = 10.83; SD = 2.89) and Picture Concepts (10.83; SD = 2.48) subtests (see Table 3 and Figure 2). From the performance profiles of the BA and CT group, it is evident that these children also perform comparatively weaker in the subtest Picture Memory (BA: M = 9.45; SD = 2.70; control: M = 9.66; SD = 2.66). Children in the BA group scored best on the Information subtest (M = 11.68; SD = 2.42), whereas children in the CT group scored best on the Bug Search subtest (M = 12.69; SD = 2.32) (Figure 2). The results of the post-hoc analyses show significant differences between the DCD and CT group for the subtests Block Design (*p* = 0.006, d = −0.97), Bug Search (*p* =< 0.001, d = −1.24) and Cancellation (*p* = 0.014, d = −0.87) as well as for children with DCD and the BA group in Block Design (*p* = 0.005, d = −1.10). In addition, children in the BA group performed worse on the Bug Search subtest than children in the CT group (*p* = 0.037, d = 0.037) (see Appendix B).

## 4. Discussion

### 4.1. WPPSI-IV Profiles of Children with Movement Difficulties (Question 1)

The aim of the present study was to investigate the extent to which deficits in cognitive abilities of children with movement disabilities described in the literature can be measured by the WPPSI-IV intelligence test battery. In this regard, not only children with DCD but also children with below-average motor development should be examined. However, if children with motor underachievement, which according to the international classification (DSM-5 [4]) is not considered a developmental disorder in the strict sense, also show tendencies toward below-average cognitive performance, the WPPSI-IV would be an economical tool for early diagnosis. Based on these performance profiles, affected children could then be offered individualized support services. 

The assumption that children with DCD exhibit visual perception deficits, which are manifested by deviating test performance in the primary index Visual Spatial, could initially be confirmed in comparison with the CT group. A more differentiated analysis at subtest level also indicates that these are particularly due to deviating performance in the Block Design subtest. The analysis of the performance profiles also reveals that the twelve children in the DCD group achieve comparatively poor test performance here compared to the other nine subtests of the WPPSI-IV. This seems logical against the background of the research results of Tsai et al. [16], for example, who found deficits in the visual perceptual abilities spatial relations, visual memory, visual discrimination, and figure-ground discrimination in their study, since the Block Design subtest measures precisely these functions. Reproducing the given patterns using small dice in a fixed amount of time also requires fine motor skills, so the combination of visual and motor demands appears to result in children with DCD achieving lower test performance in the Block Design than in subtests requiring only visual perceptual skills. These results are additionally consistent with the findings of other studies: Kastner et al. [28] and Jaščenoka et al. [29] were able to reveal that children with DCD show their lowest overall performance in the Block Design subtest in their WPPSI-III studies. However, it should be noted that the differences in performance in the present study with the children in the CT group are less pronounced than in the studies by Kastner et al. [28] and Jaščenoka et al. [29]. This effect can possibly be explained by the comparatively high parental educational level of the children studied: it can be assumed that parents with a medium and high educational background make use of (low-threshold) support and assistance services for their children significantly more often and that the children in the present study are better supported compared to a representative population average [35].

In the past, working memory performance could not be measured differentially with the WPPSI-III. Therefore, no studies are available from which to derive specific hypotheses about the test performance of preschool children with motor problems in the WPPSI-IV. Consideration of research studies in which children with DCD were tested with the Wechsler Intelligence Scale for Children-Fourth Edition (WISC-IV) [36] (a continuation of the Wechsler scales for school-age children) is also only of limited help: although various studies have shown that children with DCD perform less well on the Working Memory index than children with no motor problems, the test items of the WISC-IV tend to require auditory memory skills, whereas the WPPSI-IV is a more visual working memory test. However, the results of Leonard et al. can be used as a guide [19] that speaks for the conspicuousness of children with coordination problems in auditory working memory performance. Chen et al. [21] also reported difficulties in auditory and visual everyday memory performance. Consideration of the present descriptive results of this study alone suggests slightly reduced visual working memory performance of children with DCD compared to developmentally normal children and is thus consistent with the cited results of Leonard et al. [19] and Chen et al. [21]. It must be mentioned, however, that these differences in performance are not significant compared to the CT group: comparing all groups, it can be found that the performance between the three groups is significantly more homogeneous in the index of working memory than in the other primary indices of the WPPSI-IV. One reason for this could be that children with DCD show stronger deficits in auditory than in visual working memory. Future studies should therefore use methods that examine both auditory and visual working memory in one sample.

In the present study, the WPPSI-IV index Fluid Reasoning turns out to be independent of the children’s motor performance. The descriptive analysis of the average intelligence performance, differentiated according to the children’s level of motor development (DCD, BA, CT group), further exhibits that children with DCD achieve their best interindividual test performance in this index. The index Fluid Reasoning comprises the two subtests Matrix Reasoning and Picture Concepts. According to their notations, both tasks primarily measure fluid intelligence, but are also based on the analysis of visual stimuli. These results, in combination with the findings from the Block Design subtest, allow us to conclude that children with DCD show difficulties in the analysis of abstract and complex visual stimuli, but that the processing of simple and concrete pictures seems unimpaired. The present results suggest that the Index of Fluid Reasoning is a suitable indicator for assessing the intelligence performance of children with DCD due to its independence from the level of motor development. The WPPSI-IV authors’ recommendation to estimate the intelligence level of motor-impaired children increasingly on the basis of the Index Fluid Reasoning can be confirmed by the present results.

The significant group differences in the index Verbal Comprehension could be considered a specific sample effect at this point due to the good performance of the children with below-average and unremarkable motor performance in this index. The children with DCD achieve an average IQ of 101.08 points and thus even achieve a value that minimally exceeds the average value of 100 to be expected in an overall population. The differentiated examination of the subtests Information and Similarities of the index of Verbal Comprehension also suggests a low association of motor and language performance: On the subtest level, no performance differences between the three groups could be detected. Thus, the primary index Verbal Comprehension as well as the corresponding subtests also tend to be suitable for estimating the cognitive performance of children with DCD. 

The revision of the subtests of the WPPSI-IV in the index Processing Speed was carried out, as already described in detail in the introduction, with the aim of reducing (fine) motor requirements and thus creating a measure of cognitive processing speed. The extent to which this was successful cannot be clearly answered on the basis of the present results. First of all, it can be stated that the children with DCD achieved poorer test performance on average in the Processing Speed index than in the other four primary indices. However, if other research is taken into consideration, it can be found that these effects are comparatively smaller. It thus seems possible that the group differences between children with DCD and the CT group can be attributed less to reduced motor performance, but rather as a sign of slower cognitive processing. This hypothesis can be supported, for example, by the study results of Michel et al. [20]. They reported that children with lower motor performance show lower scores on cognitive-executive tasks under time pressure than an unremarkable CT group, which is why the authors suspect a slower basal processing speed in these children. 

Considering the Full-Scale IQ, this indicates the clearest differences between children with DCD and the BA and the CT group. This underlines the clinical-practical importance of assessing the “actual” intelligence level of children with DCD using the motor-independent indices Verbal Comprehension and Fluid Reasoning. 

### 4.2. Group Differences between Children with Movement Abilities below Average and a Control Group (Question 2)

In addition to the primary question of the extent to which the WPPSI-IV can depict cognitive deviations of children with DCD, the present study further aimed to investigate whether the intelligence performance of children with below-average motor skills differs from that of a CT group. Looking at the results of the post-hoc analyses, first of all children with below-average motor skills do not differ significantly from either the group of children with DCD or the CT group (with the exception of the Bug Search subtest after post-hoc procedure with *t*-test). However, the interpretation of these results is limited by the small sample sizes, so that the analyses of the descriptive statistics and effect sizes are used here for support. The following trend can be found: the test results achieved in the five indices of the WPPSI-IV as well as in the overall scale increase with improving motor ability, but this effect is less pronounced between the BA and CT groups. The following hypothesis is derived from this with reservations: the cognitive development of children with DCD, i.e., an actual developmental disorder, proceeds differently from that of children with unremarkable motor skills (BA and CT group), regardless of how well their motor skills are developed. This assumption is also shared by other research groups. For example, Dewey and Bernier [37] extensively address the question of atypical brain development in children with DCD. Based on current genetic and imaging findings, the authors conclude that DCD, which in addition to impairments in motor skills is associated particularly with abnormalities in various neuropsychological functions (especially in visual perception and executive functions), could be attributable to atypical brain development. 

### 4.3. Limitations

Various aspects can be named as limitations of this study. The greatest limiting factor is the selectivity of the sample. No children with a lower parental educational background were included. All parents had at least a secondary school leaving certificate, more than 80% of the parents had a technical college or university entrance qualification or even a technical college or university degree. An analysis of the descriptive data additionally indicates that the children with below-average motor performance as well as the children of the CT group achieved slightly above-average test performance in almost all indices. Furthermore, the small sample sizes can be criticized. Against the background of these two critical points, the representativeness of the present results is thus limited. 

As a final point of criticism, it should be noted that the LoMo 3-6 can only be used to calculate an overall motor skills score. A more differentiated examination of the correlations of fine motor deficits would be desirable, especially for the results of the index Processing Speed, in order to be able to determine more precisely whether the below-average test performance of the children with DCD is rather due to a reduced cognitive processing speed or is caused by low fine motor performance.

In conclusion, the results of this study can be summarized as follows:

1 Children with DCD exhibit significantly lower scores in the primary indices Visual Spatial and Processing Speed as well as in Full-Scale IQ.
2 The recommendations of the authors of the WPPSI-IV to assess the intelligence performance of children with DCD using the test scores of the Verbal Comprehension and Fluid Reasoning indices in addition to the overall IQ are supported in tendency by the present data.
3 The results for the Working Memory index cannot be interpreted unambiguously: the children with DCD achieved worse test scores here in comparison with the Verbal Comprehension, Visual Spatial and Fluid Reasoning indices, but the comparison with the CT group did not prove significant. Further research with larger and more representative samples would therefore be indicated.
4 Children with below-average motor performance, but not requiring motor therapy, do not differ from children in the CT group in terms of their cognitive performance profile.

## 5. Conclusions

The assessment of the cognitive performance level plays an essential role in the diagnosis of developmental disorders. A DCD exists when a child’s performance in a specific developmental test deviates significantly from their general intelligence level and a reduction in intelligence can be ruled out. In this context, the WPPSI-IV might be a valid and economical instrument for diagnosing related deficits in cognitive abilities in children with DCD at preschool age and, at the same time, for checking diagnostic criterion III (exclusion of intelligence impairment) according to DSM-5 [4]. Even though children with below-average motor performance tended not to show any abnormalities in the various indices of the WPPSI-IV, it makes sense to raise awareness among educational professionals and parents regarding the links between motor and cognitive development: early and comprehensive developmental support helps children to cope with the transition from kindergarten to primary school and thus set a positive course for success at school. However, due to the small sample size, these recommendations should not be generalized. Further studies on larger samples are needed to support the findings of this study.

## Figures and Tables

**Table 1:** Demographic description of the sample by group.

	Engine Performance				Group Differences
	Total (n = 66)	DCD (n = 12)	BA (n = 22)	Control (n = 32)	
Sex					0.376 ^a^
female	32 (48.5%)	4 (33.3%)	12 (54.5%)	18 (43.8%)	
male	34 (1.55%)	8 (66.7%)	10 (45.5%)	14 (43.8%)	
Age in months					0.907 ^b^
M	61.73	62.58	61.86	61.31	
SD Range	8.19 48–81	10.237 52–81	5.70 52–72	9.00 48–79	
Parental educational level					0.502 ^a^
Lower education level	0 (0%)	0 (0%)	0 (0%)	0 (0%)	
Medium educational level	12 (8.21%)	1 (8.3%)	6 (27.3%)	5 (15.6%)	
High educational level	16 (24.2%)	2 (16.7%)	6 (7.32%)	8 (25.0%)	
Highest educational level	38 (57.6%)	9 (75.5%)	10 (45.5%)	19 (59.4%)	
Migration background					0.196 ^a^
No Migration Background	52 (78.8%)	8 (66.7%)	20 (90.9%)	24 (75.0%)	
With Migration Background	14 (1.22%)	4 (33.3%)	2 (9.1%)	8 (25.0%)	
LoMo 3-6 (PR)					<0.001 ^b^
M	49.30	8.50	39.95	71.03	
SD	25.68	6.50	7.03	11.83	
Range	1–94	1–19	26–50	53–94	

Note: DCD = children with DCD or risk of DCD, BA = children with movement abilities below average, control = children without movement disabilities, a = *p*-value of χ², b = *p*-value retrieved from Kruskal–Wallis, parental education is defined as the highest level of education achieved by both parents (low educational level = no diploma or school certificate after 9th grade, medium educational level = school certificate after 10th grade, high educational level = university entrance qualification/certificate after 12th or 13th grade, and highest educational level = college/university degree), and migration background is indicated when either the child or at least one parent was not born in Germany.

**Table 2:** Descriptive statistics and characteristic values of the one-factor ANOVA for the primary WPPSI-IV index scores (IQ scores).

WPPSI-IV Primary Index Scales and Full Scale	Description						ANOVA				Post-Hoc (See Also Appendix B)
	DCD (n = 12)		BA (n = 22)		CT (n = 32)						(*p* < 0.05)
	M	SD	M	SD	M	SD	F	df	p	η^2^	
Verbal Comprehension	101.08	11.39	110.50	13.88	112.13	11.83	3.530	2	0.035 *	0.101	DCD = BA, DCD < CT, BA = CT
Visual Spatial	98.92	12.82	107.55	11.93	110.47	11.54	4.113	2	0.021 *	0.115	DCD = BA, DCD < CT, BA = CT
Fluid Reasoning	104.83	13.19	107.50	12.57	107.78	16.79	0.180	2	0.836	0.006	DCD = BA = CT
Working Memory	95.92	11.97	100.73	13.410	101.84	10.680	1.100	2	0.339	0.034	DCD = BA = CT
Processing Speed	95.75	13.78	104.59	12.831	111.16	10.417	6.431	2	0.003 **	0.170	DCD = BA, DCD < CT, BA = CT
Full Scale	98.67	12.21	108.09	9.586	110.82	10.19	6.192	2	0.004 **	0.164	DCD < BA, DCD < CT, BA = CT

Note: M = Mean, SD = Standard deviation, F = F-Test, p = Level of significance, η = Effect2 size Eta-Square, DCD = Risk of Developmental Coordination Disorder, BA = Motor performance below average, CT = Control group, * = Significant result at the 0.05-significance level, ** = Significant result at the 0.01-significance level.

**Table 3:** Descriptive statistics and characteristic values of the one-factor ANOVA for the WPPSI-IV (IQ) subtests (scaled scores).

WPPSI-IV Subtests of Primary Index Scales	Description						ANOVA				Post-Hoc (See Also Appendix B)
	DCD (n = 12)		BA (n = 22)		CT (n = 32)						(*p* < 0.05)
	M	SD	M	SD	M	SD	F	df	p	η^2^	
Verbal Comprehension											
Information	9.92	2.31	11.68	2.42	11.75	2.31	2.905	2	0.062	0.076	DCD = BA = CT
Simalarities	10.67	2.15	12.14	2.85	12.59	2.37	2.590	2	0.083	0.073	DCD = BA = CT
Visual Spatial											
Block design	8.83	2.21	11.36	2.44	11.47	2.87	4.792	2	0.012 *	0.132	DCD < BA, DCD < CT, BA = CT
Object Assembly	10.75	2.73	11.14	2.44	12.03	2.23	1.627	2	0.205	0.049	DCD = BA = CT
Fluid Reasoning											
Matrix Reasoning	10.83	2.89	11.00	2.76	11.34	2.94	0.175	2	0.840	0.006	DCD = BA = CT
Picture Concepts	10.83	2.48	11.59	2.48	11.28	3.42	0.253	2	0.778	0.008	DCD = BA = CT
Working Memory											
Picture Memory	8.75	2.83	9.45	2.70	9.66	2.66	0.492	2	0.614	0.015	DCD = BA = CT
Zoo Locations	9.92	2.07	10.77	3.21	10.94	2.18	0.712	2	0.494	0.022	DCD = BA = CT
Processing Speed											
Bug Search	9.75	2.53	11.23	2.65	12.69	2.32	6.683	2	0.002 **	0.175	DCD = BA, DCD < CT, BA < CT
Cancellation	8.75	2.53	10.32	2.50	10.69	2.10	3.089	2	0.053	0.089	DCD = BA, DCD < CT, BA = CT

Note: M = Mean, SD = Standard deviation, F = F-Test, p = Level of significance, η = Effect2 size Eta-Square, DCD = Risk of Developmental Coordination Disorder, BA = Motor performance below average, CT = Control group, * = Significant result at the 0.05-significance level, ** = Significant result at the 0.01-significance level.

## Data Availability

Since the research is based on health service research (Versorgungsforschung) the data are not publicly available.

## Appendix A

Non-parametric group comparisons of the primary indices and subtests scores of the WPPSI-IV (Kruskal–Wallis and Mann–Whitney U) are presented.

**Table A1:** Mean and standard deviation for all primary WPPSI-IV indices by group and results of Kruskal–Wallis test and Mann–Whitney U.

WPPSI-IV Primary Index Scales and Full Scale	Description						Kruskal–Wallis			Post-Hoc (Mann–Whitney U)
	DCD (n = 12)		BA (n = 22)		CT (n = 32)					(*p* < 0.05)
	M	SD	M	SD	M	SD	H	df	p	
Verbal Comprehension	101.08	11.39	110.50	13.880	112.13	11.83	6.78	2	0.034 *	DCD = BA, DCD < CT, BA = CT
Visual Spatial	98.92	12.82	107.55	11.931	110.47	11.54	6.92	2	0.031 *	DCD = BA, DCD < CT, BA = CT
Fluid Reasoning	104.83	13.19	107.50	12.573	107.78	16.79	0.456	2	0.796	DCD = BA = CT
Working Memory	95.92	11.97	100.73	13.410	101.84	10.68	2.591	2	0.274	DCD = BA = CT
Processing Speed	95.75	13.78	104.59	12.831	111.16	10.42	10.01	2	0.007 **	DCD = BA, DCD < CT, BA = CT
Full Scale	98.67	12.21	108.09	9.586	110.82	10.19	9.472	2	0.009 **	DCD< BA, DCD < CT, BA = CT

Note: M = Mean, SD = Standard deviation, H = H-Test, p = Level of significance, DCD = Risk of Developmental Coordination Disorder, BA = Motor performance below average, CT = Control group, * = Significant result at the 0.05-significance level, ** = Significant result at the 0.01-significance level.

**Table A2:** Mean and standard deviation for all subtests of the primary WPPSI-IV indices by group and results of Kruskal–Wallis test and Mann–Whitney U.

WPPSI-IV Subtests of Primary Index Scales	Description						Kruskal–Wallis			Post-Hoc (Mann–Whitney U))
	DCD (n = 12)		BA (n = 22)		CT (n = 32)					(*p* < 0.05)
	M	SD	M	SD	M	SD	H	df	p	
Verbal Comprehension										
Information	9.92	2.31	11.68	2.42	11.75	2.31	5.132	2	0.077	DCD = BA = CT
Simalarities	10.67	2.15	12.14	2.85	12.59	2.37	5.565	2	0.062	DCD = BA = CT
Visual Spatial										
Block design	8.83	2.21	11.36	2.44	11.47	2.87	9.346	2	0.009 *	DCD < BA, DCD < CT, BA = CT
Object Assembly	10.75	2.73	11.14	2.44	12.03	2.23	4.958	2	0.271	DCD = BA = CT
Fluid Reasoning										
Matrix Reasoning	10.83	2.89	11.00	2.76	11.34	2.94	0.248	2	0.883	DCD = BA = CT
Picture Concepts	10.83	2.48	11.59	2.48	11.28	3.42	1.053	2	0.591	DCD = BA = CT
Working Memory										
Picture Memory	8.75	2.83	9.45	2.70	9.66	2.66	1.406	2	0.495	DCD = BA = CT
Zoo Locations	9.92	2.07	10.77	3.21	10.94	2.18	1.734	2	0.420	DCD = BA = CT
Processing Speed										
Bug Search	9.75	2.53	11.23	2.65	12.69	2.32	10.35	*2*	0.006 **	DCD = BA, DCD < CT, BA < CT
Cancellation	8.75	2.53	10.32	2.50	10.69	2.10	2.612	*2*	0.084	DCD = BA, DCD < CT, BA = CT

Note: M = Mean, SD = Standard deviation, H = H-Test, p = Level of significance, DCD = Risk of Developmental Coordination Disorder, BA = Motor performance below average, CT = Control group, * = Significant result at the 0.05-significance level, ** = Significant result at the 0.01-significance level.

## Appendix B

Post-hoc group *t*-test comparisons for scales with significant ANOVA effects are presented. Effect sizes were classified according to Cohen (small effect d = 0.20, medium effect d = 0.50 and large effect d = 0.80 (Cohen, 1992) [38].

**Table A3:** Post-hoc group *t*-test comparisons for scales with significant ANOVA effects.

WPPSI-IV Primary Index Scales and Full Scale	DCD vs.BA			DCD vs. CT			BA vs. CT		
	T (32)	p	d	T (42)	p	d	T (52)	p	d
Verbal Comprehension	−2.018	0.052	−0.72	−2.800	0.008 *	−0.94	−0.46	0.646	−0.12
Visual Spatial	−1.964	0.058	−0.71	−2.870	0.006 **	−0.97	−0.90	0.371	−0.20
Processing Speed	−1.871	0.071	−0.67	−3.703	<0.001 **	−1.25	−1.72	0.091	−0.47
Full Scale	−2.486	0.018 *	−0.89	−3.380	0.002 **	−1.14	−1.10	0.278	−0.30

Note: T = *t*-test, p = Level of significance, d = effect size Cohens’d, DCD = Risk of Developmental Coordination. Disorder, BA = Motor performance below average, CT = Control group, * = Significant result at the 0.05-significance level, ** = Significant result at the 0.01-significance level.

**Table A4:** Post-hoc group *t*-test comparisons for subtests with significant ANOVA effects.

WPPSI-IV Subtests of Primary Index Scale	DCD vs.BA			DCD vs. CT			BA vs. CT		
	T (32)	p	d	T (42)	p	d	T (52)	p	d
Block design	−2.983	0.005 *	−1.10	−2.867	0.006 **	−0.97	−0.140	0.889	−0.04
Bug Search	−1.577	0.125	−0.57	−3.652	<0.001 **	−1.24	−2.143	0.037 *	−0.59
Cancellation	−1.743	0.091	−0.63	−2.577	0.014 *	−0.87	−0.588	0.559	−0.16

Note: T = *t*-test, p = Level of significance, d = effect size Cohens’d, DCD = Risk of Developmental Coordination. Disorder, BA = Motor performance below average, CT = Control group, * = Significant result at the 0.05-significance level, ** = Significant result at the 0.01-significance level.
